# Could we predict an episode of delirium tremens? Case report

**DOI:** 10.1192/j.eurpsy.2023.1364

**Published:** 2023-07-19

**Authors:** A. Tounsi, Z. Bencharfa, F. Azraf, M. Sabir, F. Elomari

**Affiliations:** 1 psychiatry; 2 addiction, Arrazi university psychiatric hospital, salé, Morocco

## Abstract

**Introduction:**

Delirium tremens is one of the most serious complications associated with alcohol withdrawal. It affects a percentage of 5 to 20% of users and is not related to the duration of consumption nor to the quantities taken. An early diagnosis will facilitate a quick treatment without putting at risk the vital prognosis.

**Objectives:**

Our objective is to identify the different indicators mentioned in the existing literature and to compare these to the clinical and paraclinical data of our patients

**Methods:**

We present through clinical vignettes, the cases of two patients hospitalized in our department of addictology for a cure of alcohol withdrawal and who presented an episode of delirium tremens.

**Results:**

everal clinical and paraclinical parameters have been linked to statistically significant differences in the published reports related to this subject. Thrombocytopenia remains the common element between the different publications and was the case in our two patients.

Clinically, the presence of a previous episode of delirium or seizure during withdrawal , as well as tachycardia (>100 bpm) and low number of quit attempts were significantly related to the occurrence of delirium tremens. The majority of the predictors identified were paraclinical and included: hyponatremia, hypokalemia, elevated ALT and homocyctein levels, low pyridoxine levels, and the presence of structural brain damage.

**Conclusions:**

the literature on predictors of delirium tremens remains poor. more studies are needed to confirm the data already mentioned

**Disclosure of Interest:**

None Declared

